# Health in the 'hidden population' of people with low literacy. A systematic review of the literature

**DOI:** 10.1186/1471-2458-10-459

**Published:** 2010-08-05

**Authors:** Phyllis Easton, Vikki A Entwistle, Brian Williams

**Affiliations:** 1 NHS Tayside, King's Cross, Clepington Road, Dundee, UK; 2 Social Dimensions of Health Institute, Universities of Dundee and St Andrews, 11 Airlie Place, Dundee, UK

## Abstract

**Background:**

Much of the evidence of an association between low functional or health literacy and poor health comes from studies that include people who have various cognitive difficulties or who do not speak the dominant language of their society. Low functional or health literacy among these people is likely to be evident in spoken conversation. However, many other people can talk readily about health and other issues but have problems using written information. Consequently, their difficulties may be far less evident to healthcare professionals, creating a 'hidden population' whose functional or health literacy problems have different implications because they are less likely to be recognised and addressed.

We aimed to review published research to investigate relationships between low functional or health literacy and health in working age adults who can converse in the dominant language but have difficulty with written language.

**Methods:**

We searched reviews and electronic databases for studies that examined health-related outcomes among the population of interest. We systematically extracted data relating to relationships between low functional or health literacy and both health status and various possible mediators or moderators of the implications of literacy for health. We developed a narrative review.

**Results:**

Twenty-four studies met our inclusion criteria. Lower functional or health literacy in this population was found to be associated with worse health status. This may be mediated by difficulties accessing healthcare, and poorer self-management of health problems. It is currently unclear whether, how or to what extent these difficulties are mediated by poorer knowledge stemming from low functional or health literacy. The variation in functional or health literacy measures and comparisons make it difficult to compare study findings and to establish the implications of different literacy issues for health outcomes.

**Conclusions:**

There is evidence in the literature that low functional or health literacy is associated with poor health in the 'hidden population' of adults whose literacy difficulties may not be evident to health care providers. Further research is needed to help understand the particular disadvantages faced by this population and to establish appropriate responses.

## Background

Despite economic and social growth, low literacy continues to be a significant issue across the developed world. The 1996 OECD International Adult Literacy Survey found 22% of US adults and 23% of UK adults to be performing at the lowest level of a 5-point scale of functional literacy [1]. Functional literacy was defined for this survey as *"the ability to read, write and speak in English, and to use mathematics at a level necessary to function at work and in society in general" *[1].

In healthcare contexts, the concept of health literacy is often preferred. Health literacy has been defined as *"the cognitive and social skills which determine the motivation and ability of individuals to gain access to, understand and use information in ways which promote and maintain good health" *[2]. Low levels of health literacy have also been reported in a number of contexts [3-6].

Both low functional literacy and low health literacy may contribute to poor health status through a variety of mediators and moderators [7]. They may contribute (perhaps via reduced ability to use written instructions and advice) to reduced adherence to effective medication regimes and to serious medication errors [8]. Low functional literacy may affect health via its negative implications for social functioning and social status (including social stigma) [9]. Low health literacy is likely to be associated with limited knowledge of health and healthcare issues, which may contribute to poor self-management of long-term conditions [10-12].

It is difficult to differentiate between the implications of low functional literacy and low health literacy. There is clearly an overlap between the two concepts, but the relationship between them is not simple. While low functional literacy is very likely to impede the achievement of high health literacy, a person may have high functional literacy but low health literacy. The measures of health literacy that are currently available do not assess all aspects of the concept [13] and much of what they do assess could be considered relevant to functional literacy as well. For example, the REALM focuses primarily on *reading ability *and does not examine motivation, understanding, or ability to access or use health-related information in any detail. In practice, then, assessments of health literacy are closely linked to assessments of functional literacy. People who are functionally literate are likely to score high on the existing measures of health literacy even though they may have low health literacy according to the definition given.

The implications of low functional or health literacy for health are likely to be mediated or moderated by a number of factors, including the extent to which health services and health professionals recognize and make allowances for these. Low functional or health literacy is likely to be more readily recognized in some groups than others. For example, it will be evident in healthcare consultations that people from minority ethnic groups who do not speak the dominant language of their country of residence or health service will have functional and health literacy difficulties in that context unless alternative language provision is made. When literacy difficulties are associated with language differences, they may be addressed through interpretation and translation. Health professionals may also be more likely to consider the possibility of literacy difficulties among older than younger adults if they associate ageing with visual and/or cognitive impairments, or think that older cohorts were more likely to have missed out on schooling as children. They might thus be more likely to give clear or increased oral instruction to older people.

In developed countries with a compulsory education system, there may be a large 'hidden population' of people with literacy difficulties. The ability to communicate orally can mask the inability of many people who speak the dominant language well to read and write competently. Some of these people are unaware they have low health literacy skills [1] and many are reluctant to disclose them and careful to use coping strategies that hide them [9]. Several studies have shown that health care staff often do not recognise health literacy difficulties among working age adults who can engage in spoken conversations in the dominant language [14-16].

Much of the research that has established associations between low functional or health literacy and health status to date has included several or all of the groups of people whose literacy difficulties are more likely to be recognised and addressed by health services and staff. This may obscure important differences in the ways in which literacy can affect health. Relatively little is known about the implications of low functional or health literacy among those people whose literacy difficulties are more hidden. Specific attention to this group may be important to support the development of strategies for reducing any adverse health implications of their literacy difficulties.

The current review was developed to further our understanding of the relationship between functional or health literacy level and health in a working age population whose low functional or health literacy skills may be neither obvious nor readily identifiable to health care staff and others.

## Methods

The review sought to establish evidence of associations between low functional or health literacy and health in a working age population whose first language was the dominant language of their country. We considered relationships between measured literacy and health status, and considered how these might be mediated or moderated by attending as well to relationships between measured literacy and a variety of health-related behaviours and activities.

### Review questions

The review questions were, for the population of interest:

1. What evidence is there of an association between functional literacy or health literacy level and health status?

2. What evidence is there of an association between functional literacy or health literacy level and the following potential mediating variables:

• health promoting or health risk behaviours?

• access to and use of health services?

• self-management of health problems?

3. Is there evidence that knowledge of particular health risk or health conditions may mediate the relationship between functional literacy or health literacy and health behaviours?

### Inclusion Criteria

We considered studies of any design which examined relationships between functional literacy or health literacy (assessed by a validated measure or recognised by attendance at an adult literacy program) and health outcomes or health-related knowledge or behaviours in a working age population whose first language was the dominant language of their resident country. A full list of inclusion and exclusion criteria is included in Additional file 1, Table S1.

### Search strategy

We searched for relevant studies in two stages, looking first at studies that had been included in previous, readily identifiable reviews of functional or health literacy and then applying a sophisticated supplementary search strategy to relevant electronic databases.

The first search for readily identifiable reviews was conducted using the key terms "health" AND "literacy" AND "review" in MEDLINE, CINAHL, British Nursing Index, EMBASE, ERIC and PsycINFO.

The second search strategy, which was used to check for any relevant studies that had not been included in previous reviews, covered the following databases:

MEDLINE 1950 - December 2008; CINAHL 1982 - December 2008; British Nursing Index 1994 - December 2008; EMBASE 1980 - December 2008; ERIC 1965 - December 2008; PsycINFO 1967 - December 2008; and ASSIA 1987 - December 2008.

The search comprised key terms associated with the inclusion criteria, tailored for each electronic database. Although some search terms were common to all search strategies, adjustments were made to take advantage of the different indexing terms available within individual databases, and to add a health focus to those databases that did not have this by default.

In order to ensure that as many studies as possible were identified, the search strategy was designed to have high sensitivity even though this would likely be at the cost of reduced specificity. A key contributor to this was the decision to include studies indexed by the term 'educational status' even though most of these studies related to years of schooling. Abstract appraisal was carried out by PE; VE and BW appraised a 10% random sample. Full text appraisal was carried out by PE and one third of papers retrieved for full text appraisal were assessed by all three reviewers; VE and BW carried out independent appraisal of any other papers about which there was uncertainty over inclusion.

### Data extraction

Data relating to study design, populations, sampling, functional or health literacy levels and health outcomes were systematically extracted from each paper by PE. Key findings are presented in Additional file 2, Table S2. Differences were considered to be statistically significant at p < .05. Statistically significant findings are reported numerically; otherwise, results are reported as not significant, even if the authors of the particular study considered this level to be statistically significant. We extracted data relating to the following indicators of research quality: response rate; whether the person measuring the health outcome was blinded to participants' health literacy scores; whether confounding was addressed.

Due to the highly diverse nature of populations and health outcomes investigated, differing health literacy measures and cut-off points to make comparisons, we undertook a narrative synthesis of findings.

## Results

A total of 24 relevant papers were included in the review. The initial stage of the search strategy identified four reviews that focused on health outcomes among our age groups of interest [17-20]. Of the 57 primary studies included within these four reviews, 11 met our inclusion criteria.

**Figure 1:**
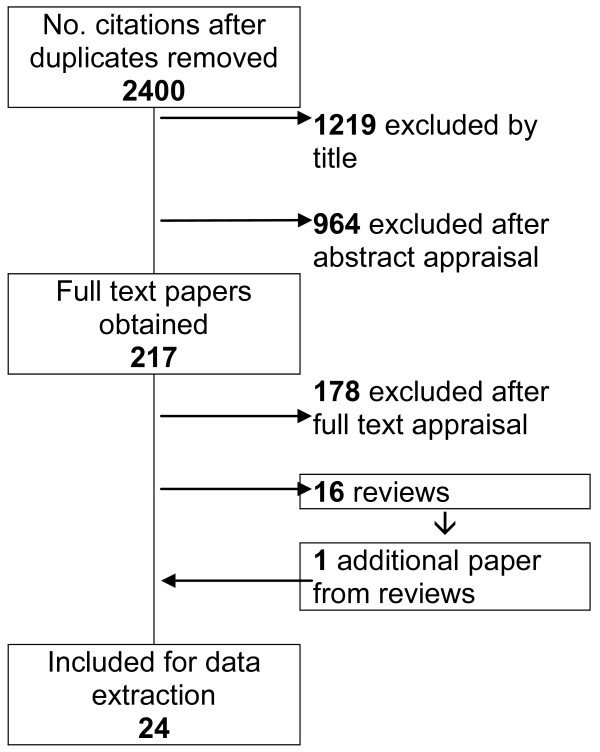
Identification of studies

The second stage of the search strategy identified 2400 citations. Figure 1 shows the number of documents excluded at each stage. Additional reviews identified at this stage were also searched for primary papers not appearing in the citations from the database searches. Exclusions at full text appraisal stage were largely due to studies not meeting our criteria for age or ability to speak the dominant language.

The 11 studies identified from the initial reviews were also identified by the second stage search. The 2400 citations from the second stage search yielded an additional 13 studies that met our inclusion criteria.

The twenty-four papers that were included reported mainly on studies conducted in the USA (two were from the UK and one from Canada) (Additional File 2, Table S2). Studies used diverse methods to investigate the implications of functional or health literacy for various health-related issues in a range of health care contexts. The health related issues that the twenty-four papers considered were grouped into one or more of five outcome categories reflecting the five areas of interest in the review questions. An additional category of 'emotional responses' that it seemed inappropriate to treat simply as examples of (end state) health status emerged during the process and has been included (Additional file 2, Table S2).

Three measures of health literacy (REALM, TOFHLA and s-TOFHLA) and two measures of functional literacy (NART and the Test of Basic Adult Education) were used across the 24 papers included in the review (Additional file 2, Table S2). Implementation of the measures and cut-off points to determine low health literacy differed even when the same measure was used. (Additional file 2, Table S2).

All but three papers [21-23] considered potentially confounding demographic factors in their analysis. Only two papers [24,25] clearly reported that the person who assessed health data was blinded to study participants' health literacy status. We now summarise the key findings relating to each of the five health-related outcome domains.

### Relationship between functional or health literacy and health status

There is some evidence from 3 cross-sectional studies that lower functional or health literacy is associated with poorer health status, assessed by self-report or more objectively [26-28].

Studies of 1892 emergency department walk-in patients and of 339 people living with HIV-AIDS both found that those with lower health literacy were significantly more likely to self-report their health as poor [26,27]. In the study of people with HIV-AIDS, recorded CD4 cell counts and undetectable viral loads in the medical notes confirmed the poorer health status of those with lower health literacy [27]. Both studies used the TOFHLA to measure health literacy but compared different cut-off points: Baker et al. [26] compared the highest and lowest of three health literacy levels (0-59 vs. 75-100) while Kalichman et al. [27] compared those above and below 80% correct.

A third study measured reading level using the Test of Basic Adult Education in 193 adult learners. Those with very low reading levels (at or below 4^th ^grade), had significantly lower scores on the physical and psychosocial domains of the Sickness Impact Profile than those with higher (5^th ^grade+) reading levels [28]. This study used an objective measure of health but focused on a group of people who were motivated to address their literacy difficulties and so were not necessarily representative of the general population with low literacy. People who have sought help with literacy may be more likely to let health professionals know they have difficulty with reading and writing. Psychosocial health impairment may be more prevalent in those who do not seek help with literacy education and so may be underestimated by this study.

### Relationship between functional or health literacy and health promoting or health risk behaviours

Five studies were found to have investigated the relationships between health literacy levels and preventive health or health risk behaviours [22,29-32]. All used the REALM to measure health literacy but no two used the same levels for comparison. Findings from these studies were complex and mixed.

Two studies found some higher health risk behaviours in those with lower health literacy but also some potentially conflicting evidence [22,29]. In a US study of 130 women referred for colposcopy after abnormal pap smear, those with higher health literacy reported a greater number of risk factors for cervical cancer. Differences for individual risk factors varied; those with higher health literacy were more likely to report oral contraceptive use and having had 5 or more sexual partners in total while those with lower health literacy had higher parity. Health literacy was not associated with intercourse aged ≤18 years or with history of sexually transmitted disease other than HPV [29]. One UK study of 505 family planning clinic users, found that women with lower health literacy were: more likely to have been aged under 16 at first sexual intercourse; less likely to have used contraception at that time; and more likely to have had two or more partners in the previous 6 months [22]. The UK study of family planning clinic users found no significant difference across health literacy levels in planned or unplanned pregnancies, previous use of emergency hormonal contraception or number of sexual partners in the previous four weeks [22].

Comparison of these two studies is difficult because they categorised health literacy levels differently. Although both used the REALM, in the US study, participants fell into a broad range of health literacy levels and those scoring below 9^th ^grade were compared with those at 9^th ^grade or above [29]. The UK study converted the scores to UK reading ages; all participants had a reading age of 12 and above and comparisons were made between those with a reading age of 12-14 and 15+ [22]. This may explain some of the variance in the evidence; however, the studies also differed in the age by which first sexual intercourse was reported and the time period over which previous sexual partners were reported. Sexual health behaviours may also have been subject to different cultural influences in the two study settings. Multivariate analysis was not carried out in the UK study but the authors of the US study considered years of education, knowing someone with cervical cancer and having previous colposcopy as potential confounding factors.

Multivariate analysis from two further US studies found no association between health literacy and health risk behaviour. In a study of 600 pregnant women, no correlation was found between reading level and smoking prevalence [30]. In this group, race was significantly associated with smoking practice, African American women being significantly less likely to smoke and being significantly more likely to have lower health literacy. In a study of 423 female prison inmates, many of whom had dropped out of school, HIV risk behaviour was associated with educational attainment but not with health literacy [31]. One of the studies [31] compared 3 and the other [30], 4 health literacy levels across the REALM score.

In a further US study, lower health promoting behaviour in those with low literacy did not reach statistical significance. A cross-sectional study of 61 new mothers found that those with lower health literacy were less likely to initiate and sustain breastfeeding for the first two months of their infant's life. Breast feeding for at least two months was associated, but not significantly, with higher (12^th ^grade+) health literacy [32]. This study had an insufficient number of participants and only two literacy categories, 7^th^-8^th ^grade and 12^th ^grade+ and this may have contributed to the lack of statistical significance.

This small group of 5 studies did not produce convincing evidence of a clear association between functional or health literacy and preventive health or health risk behaviours.

### Relationship between functional or health literacy and access to and use of health services

One small qualitative study of 8 adults who participated in a community college literacy program and had been hospitalised met our inclusion criteria [33]. We assessed this study to establish what issues were identified in relation to the research questions and to ascertain whether any of these had been investigated in the quantitative studies. Participants had experienced impaired decision-making and given uninformed consent to interventions [33]. They reported having been unsure of what was expected of them as patients because they had been unable to read instructions, for example, on menus and notices [33]. They experienced fear; worry; powerlessness; stigma; vulnerability; diminished self-efficacy in accessing health services, and they balanced the risks of exposure of their literacy difficulties (stigma, decreased self-esteem) with the risks of non-disclosure (lack of knowledge gathering). Some, however, felt that the hospital was a special place where vulnerability could be shared and staff would keep information confidential. The frequency and distribution of these issues were not explored in the quantitative studies.

A further 6 quantitative studies focused on access to and use of health services. Two studies found no evidence [21,25] and one found some evidence [26] of associations between health literacy and uptake of services. One study found an association between low health literacy and poorer access to treatment [27] in some cases. Evidence of association between health literacy and relationships with healthcare staff was mixed and unclear [34,35].

A study of 543 parents found, as a secondary outcome measure, no correlation between total REALM score and accessing of preventive services for their children [25]. This could also have been considered as health promoting behaviour. A study of 202 African American women's use of prenatal care found no difference between high and low health literacy groups in the proportions of women beginning prenatal care. This study was underpowered, however, and had sought to exclude women who had no prenatal care notes [21].

Evidence from three studies suggested that low health literacy - as measured by the TOFHLA - may be associated with less appropriate use of health services or access to optimum treatment. A cross-sectional study of 1892 people attending an emergency walk-in department found that those with inadequate health literacy were more likely to have been hospitalised in the previous year than those with adequate health literacy [26] although their more frequent use of health services in general was non-significant after adjustment [26]. A study of 339 people with HIV-AIDS found those with lower health literacy were less likely to have been prescribed antiretroviral medication [27]. A further study reported an association between health literacy and relationships with healthcare staff, which may have implications for ensuring access to optimum treatment. Among 294 people living with HIV/AIDS, those with lower health literacy were no less likely to say that the doctor answered all their questions, but they were significantly less likely to say their doctors asked their opinion about treatment, or that they explained things so they could understand [35].

Collectively these 6 studies suggest that in relation to health service use, the differences between people with higher and lower levels of literacy are to be found less in terms of initial gaining of access to services and more in terms of the appropriateness of patterns of use and the securing of appropriate treatment.

Relationships with healthcare staff featured in an additional study of 157 parents of children aged one to four who had visited a well-child clinic. Those with a REALM score below 9^th ^grade reported higher quality patient-provider relationships compared to those with scores of 9^th ^grade or higher, through better family-centred care, helpfulness and confidence building [34].

### Relationship between functional or health literacy and self-management of health problems

Eight studies examined associations between functional or health literacy and aspects of self-management of manifest health problems.

Four studies used quantitative techniques to assess adherence to medication and reported a relationship between lower functional literacy or health literacy and poorer adherence, [36-39] two of them in relation to parents administering medication to their children [38,39]. A further two studies focused on parental ability to administer medication to their children [25,40]. One study investigated women's compliance with follow up treatment [24].

One qualitative study of 25 people infected with HIV investigated the perceived clarity and level of difficulty of self-report HIV medication adherence measurement tools. Patients found it difficult to define adherence, had difficulty identifying medication and in recalling missed doses [41]. These difficulties have implications both for patients' adherence and for research that seeks to investigate this.

Studies of people living with HIV/AIDS found that in a sample of 381 people, those with lower health literacy were significantly more likely to miss at least one dose of medication over a 2 day period [36] and in another study of 87 HIV+ patients, that higher health literacy was associated with 95% or greater adherence over 3 months [37]. The two studies used different health literacy measures, Kalichman et al considering low health literacy to be less than 86% correct on the TOFHLA [36] and Graham et al comparing those with a REALM score below 9^th ^grade level with those 9^th ^grade or above [37]. Both studies considered relatively small variations in adherence but findings were consistent over the two widely different timescales.

In a retrospective cohort study of 150 parents of children with asthma, those with low health literacy had used rescue medication for their children more frequently and in greater amounts. They also had a significantly greater incidence of hospitalisation and days missed from school as well as an increase in emergency department visits which approached significance [38]. In a cross-sectional study of 78 children with type 1 diabetes, glycemic control was correlated with mothers' functional literacy as measured by NART scores [39].

In one cross-sectional study of 181 parents and caregivers, those with lower health literacy, measured by the TOFHLA, reported greater use of nonstandardised dosing instruments to give their children medication and this may impact on their adherence to the medication [40]. Another study of 543 parents found no association between parents' total scores on the REALM and their ability to administer their child's medication [25].

Compliance with recommended follow-up interventions was the focus of one study of 68 women who had had an abnormal pap smear. This study considered both physicians' subjective assessments of women's health literacy and more objective measurement using the REALM. Although there was a high level of agreement between the two, only subjective physician assessment of patient health literacy was a significant predictor of failure to follow up [24].

### Knowledge of particular health risk or health conditions as a mediator between functional health literacy and health behaviours

Twelve studies focused on or included associations between functional or health literacy levels and knowledge about health conditions or treatment [25,27,35,37,38,40-42] or health risks [22,23,30,43]. Most, but not all of the studies, demonstrated lower knowledge of the various topics of interest in those with lower health literacy; two studies found that knowledge did not necessarily mediate behaviour [30] or adherence [37]. One study found that lack of knowledge was associated with behaviour likely to impact on adherence but adherence itself was not assessed [40].

Knowledge of HIV/AIDS in 372 patients offered HIV testing was poorer in those with lower health literacy [42]. One paper reported that in a sample of 294 people living with HIV/AIDS, those with lower health literacy were significantly more likely to believe that HIV transmission was less likely if anti-HIV medication was taken or if viral load was undetectable [35]. Poorer knowledge of their health status; [27,35] poorer knowledge of medication [41] and more mistaken beliefs about their treatment [37] were also reported among patients with HIV/AIDS and lower health literacy.

One study of 181 parents and caregivers found that those with lower health literacy lacked knowledge about weight-based dosing and this was associated with the use of nonstandardised medication dosing instruments [40]. Another study of 150 parents reported that low health literacy was associated with less parental asthma related knowledge, characterised by a two point difference in a 20 point questionnaire [38].

A study of 600 pregnant women reported that those with lower reading levels had lower knowledge and less concern about the health effects of smoking on their unborn babies [30]. Other studies of 406 women in the community [43] and 505 female family planning clinic patients [22] found women with low health literacy were more likely to want to know more about birth control, [43] had lower knowledge of sexually transmitted infections [22] and were less likely than those with adequate health literacy to know about fertile times within their menstrual cycle [22,43].

The 10 studies which found associations between knowledge of specific health issues and health literacy used 4 different measures and 8 different cut-off points for comparison, so although results suggest that knowledge is related to health literacy, (as would be expected, given the definition of health literacy), as with other relationships with health outcomes, it is unclear what aspects or levels of health literacy are most important.

Two studies found no association between knowledge of health issues and health literacy score. One study found that among 543 parents, knowledge of their child's diagnosis, medication name, purpose and instructions for use was not associated with health literacy score [25]. In this particular study, parents with lower health literacy considered their child more sick for the same degree of illness compared with those with higher literacy and this may have had an impact on parental management of their child's medication [25]. Another paper reported that among 400 women attending a family planning clinic, knowledge of contraception was generally poor, and although it tended to be better in those with higher health literacy, understanding of side effects of oral contraception and what to do about multiple missed pills was not associated with health literacy [23]. It is unclear why this particular study differs from the others with a similar focus examined here.

Two studies compared knowledge with related behaviour. One found that knowledge did not mediate smoking behaviour among pregnant women. Those with higher health literacy had greater knowledge but the trend was towards higher smoking in this group although the relationship was not significant [30]. Another study reported that some beliefs about medication did not mediate the relationship between health literacy and adherence and although beliefs about adherence norms were associated with adherence itself, this was independent of health literacy [37].

### Emotional responses

A further two studies focused on emotional responses of patients, either to their actual condition [29] or to scenarios related to their condition [44]. Although emotional wellbeing can be considered as a contributory indicator of health status, we have reported these studies separately because it seems important not to obscure the possibility that the 'outcomes' they report might mediate other health status changes. Among 130 women at risk of developing cervical cancer, those with lower health literacy were more likely to have excessive levels of distress [29]. In a sample of 294 people living with HIV/AIDS, those with low health literacy had greater symptoms of affective depression but less evidence of negativistic thinking; they were more likely to endorse feelings of emotional distress, lower optimism and maladaptive coping when presented with a scenario of increased viral load [44].

## Discussion

The review has identified and summarised the reported associations between low functional or health literacy and health in the 'hidden population' of people with health literacy problems. It has also considered research relating to important moderators and mediators in the relationships between literacy and health, particularly health service use and self care behaviours. However, studies have not always considered the full range of factors that may mediate or moderate the relationship between functional or health literacy and health, and some of the reported associations may obscure confounding factors.

As anticipated, there is evidence here that in the 'hidden population,' low functional or health literacy is associated with poorer health status. These associations with poorer health may be mediated through differential use of services and access to good quality treatment. For example, the higher hospitalisation rates among those with lower health literacy [26] may suggest poorer self management, patients becoming more ill, presenting at a later stage to health services or waiting until they are in crisis before they contact services. In contrast to this, one study found no difference in uptake of preventive services related to health literacy level [25]. This may be because parents with low literacy may receive information about preventive health from additional sources or because they implement strategies to cope with and ensure they do not reveal their issues with literacy. For example, one of the questions in this particular study asked whether parents knew the date of their next well child appointment. People with low literacy skills may be more likely than those with high literacy skills to memorise appointment dates (rather than rely on checking appointment letters or diaries) to ensure they keep them, and so more likely to score well on this question. The lower likelihood of access to a particular treatment such as antiretroviral medication [27] may be associated with patient-provider relationships; poorer communication or diminished self-efficacy in gaining treatment among patients with low health literacy. However, none of these issues have been studied and it is not clear whether broader aspects of health literacy such as motivation or ability to navigate the health system have been instrumental.

Evidence of poorer adherence [36,37] and poorer care of children [38-40] suggests that people with lower health literacy skills may be less likely to adopt effective health promoting or self-care behaviour. This may not be related directly or exclusively to difficulty with reading and following instructions. Subjective physician assessment of patient health literacy was a significant predictor of failure to attend follow up for treatment [24] and although there was a high correlation between their assessment and objective measures of health literacy, physicians did not correctly assess health literacy levels of all the patients. Some physicians' predictions may have been based on the assessment of other aspects, such as the degree of engagement, attitude and body language of patients. Although these findings suggest that healthcare professionals may be able to assess likely literacy problems among their patients when asked, this is not normally the case in routine practice, as reported elsewhere [14-16]. Qualitative research suggested that people with lower health literacy could struggle with some of the methods used to assess treatment adherence [41] and this may affect the measurement and reporting of adherence studies in general.

In examining differences in relationships with healthcare staff, it is not clear to what extent these findings reflect differential treatment by staff or differential perceptions of treatment by service users. It is possible that people with lower health literacy may respond differently to questions about quality of service or have different expectations from those with higher health literacy skills [34]. The study of people living with HIV-AIDS who reported differential explanations by doctors about their condition raises the issue that those with lower health literacy may also have reduced opportunities to improve their health literacy [35].

Other studies have identified a range of patient characteristics which may impact on those with low functional or health literacy accessing services and carrying out self-care activities. Poorer knowledge of health status [27,35] or medication [41] have been reported, but there is no evidence presented here that knowledge of a condition or medication has a direct relationship with adherence to treatment and two studies found that knowledge did not mediate behaviour [30] or adherence [37]. The studies of people with HIV-AIDS all recruited participants through the use of flyers or through providers, which may have led to a degree of self-selection and the results may not apply across the population living with HIV-AIDS [27,35,36,44].

Emotional responses and/or diminished self-efficacy may be mediators in the relationship between health literacy and health [29,33,44] and this may include the stigma associated with low health literacy reported elsewhere, [9] as it has been suggested that higher awareness of patient need among healthcare staff may improve patient experience [25].

This review should be interpreted in the context of several considerations. A large number of papers were initially identified. However, considerable proportions were excluded by title or after abstract appraisal. The inclusion of 'educational status,' occasionally but not frequently used to describe literacy, as a search item, increased the sensitivity of the search but also contributed greatly to the number of papers that were subsequently excluded.

Many of the studies included in this review did not state language eligibility criteria for participants and primary language has been assumed from country and ethnicity in several papers. In addition, in some of the included studies, participants were required only to be "English speaking," not to have English as a primary language. (Additional file 2, Table S2) Ethnicity, used as a proxy for language, is likely to overestimate the proportion with language difficulties, particularly in groups where there are second or third generation adults. This has probably resulted in some relevant papers being excluded and an underestimation of the effect in our population of interest. However, this was deemed more appropriate because where language was not explicitly stated we could not assume the population matched our requirements. Similarly, studies which focused on adults over the age of 65 were excluded but many people over this age do not have reduced cognitive skills which impact on literacy skills, so once more some relevant papers may have been excluded. Again, this was deemed appropriate because there was no way of distinguishing among such populations.

The studies used different cut-off points to indicate lower or higher functional or health literacy. The effect of this is unknown and it remains unclear what levels of functional or health literacy impact on health and self-care, whether and to what extent there are thresholds and/or gradient effects in different contexts and in relation to different aspects of health and healthcare. This has been highlighted as an issue across the body of health literacy research [45].

Most of the papers in the review reported on studies conducted in the US. The findings may not be generaliseable to other countries. For example, in the UK, use of the National Health Service does not require patients to engage in complex funding-related paperwork to access health services, and the reduced employment prospects associated with low health literacy would not have such significance for access. Nevertheless, the National Health Service in the UK provides most of its health advice and support for management of long term conditions in written form and patients are expected to read and implement medication instructions and drug warning labels on medicines obtained on prescription and over the counter.

Overall, the findings of associations between lower functional or health literacy and (a) poorer adherence to recommended/prescribed health care interventions and (b) poorer health are broadly similar for the 'hidden population' of people with lower health literacy as for people who may have language and/or obvious cognitive/communicative impairments in addition to health literacy difficulties [17-20]. In general, the stigma associated with low literacy skills and the coping mechanisms implemented by those with low literacy are likely to lead to refusal of many individuals from the populations of interest to participate in research. This may underestimate the prevalence or effects in some studies and particularly in the 'hidden population' we have focused on.

## Conclusions

The review has identified some evidence of association between low functional or health literacy in the 'hidden population' of people with literacy problems, but a number of important questions remain unanswered. Further research is necessary to be able to understand the difficulties faced by people within this 'hidden population' in accessing health care and in self-care activities and to identify the mediators and moderators in the relationship. Studying such a population presents many difficulties, not least in identification of individuals with functional or health literacy problems. It is important for healthcare staff to bear in mind that there is a 'hidden population' of people with low health literacy who appear to experience similar barriers to health and health services as those whose health literacy problems may be more obvious.

## Competing interests

The authors declare that they have no competing interests.

## Authors' contributions

All three authors contributed to the search, organisation and analysis of the review papers and to all stages of the writing and editing the final paper for submission.

## Pre-publication history

The pre-publication history for this paper can be accessed here:

http://www.biomedcentral.com/1471-2458/10/459/prepub

## Supplementary Material

Additional file 1 Inclusion criteria for papers. Click here for file

Additional file 2 Key findings from data extraction of review papers. Click here for file
