# Supplementary material for: Health in the 'hidden population' of people with low literacy. A systematic review of the literature
Source: BMC Public Health. 2010 Aug 5;10:459. doi: 10.1186/1471-2458-10-459 (PMC2923110; doi:10.1186/1471-2458-10-459)
Supplement: Additional file 1 — Inclusion criteria for papers. [file 1471-2458-10-459-S1.DOC]

Table 1. Inclusion criteria for papers

| ***Participants*** | *Inclusion –* At least 95% of participants age 16-65.  At least 95% whose first language is the dominant language of the study country/setting. Exclusion - Adults who do not speak the dominant language of the study country or for whom this is a second language. Adults with reduced literacy skills known to be associated with pathology, such as stroke patients, brain and head trauma patients, people with diagnosed dementia, congenital conditions, people diagnosed as having a learning disability.  Studies carried out in developing countries. |
| --- | --- |
| ***Study designs*** | Any observational study, including cross-sectional, cohort and case-control, examining relationships between measured literacy and aspects of health care or health. Any experimental study testing differences in health experiences according to literacy.  (Different literacy levels should be demonstrated in categories or on a continuum using a validated tool to measure functional literacy/health literacy/reading ability/numeracy).  Any qualitative study which explores the relationship between literacy and health.  (Qualitative data should be collected from adults with low literacy as demonstrated by testing or adults participating in basic skills education. It should examine experiences of health care and/or health). |
| ***Outcomes*** | *Self-management of health problems* - adherence; management of long-term conditions; management of acute conditions; knowledge of condition; knowledge of treatment regime Preventive health – health behaviour; attitudes; knowledge. *Access to and use of formal health services* – type, frequency and appropriateness of access; experiences of use.  *Health status* - morbidity or mortality; functional status; health-related quality of life. |
