# Supplementary material for: Health in the 'hidden population' of people with low literacy. A systematic review of the literature
Source: BMC Public Health. 2010 Aug 5;10:459. doi: 10.1186/1471-2458-10-459 (PMC2923110; doi:10.1186/1471-2458-10-459)
Supplement: Additional file 2 — Key findings from data extraction of review papers. [file 1471-2458-10-459-S2.DOC]

## Table 2. Key findings from data extraction of review papers

| **Lead author (date), country** | **Design** | **Sample size**  **and origin** | **Language** | **Functional or health literacy measure (Comparison made)** | **Outcome category(ies)** | **Outcome summary** |
| --- | --- | --- | --- | --- | --- | --- |
| Arnold CL (2001), US[30] | Cross-sectional; Face to face interviews | 600 pregnant women outpatients at obstetric clinic; Convenience and quota to ensure equal White/African American | English (assumed) | REALM  (<=3rd grade; 4th-6th grade; 7th-8th grade; 9th grade+) | Health promoting/risk behaviours;  Knowledge | Reading level not correlated with smoking prevalence in pregnant women. Women <=3^rd^ grade level less knowledgeable about potential for adverse health effects for mother and for baby and what these might be (p<.001). Low literate women least knowledge about effects of second hand smoke on children (p<.001). Women 9^th^ grade+ level more likely to be extremely concerned about health effects of smoking on their babies compared with <=3^rd^ grade (66% vs. 37%, p<.001). |
| Baker DW (1997), US[26] | Cross-sectional;  Face to face interviews | 1892 emergency department walk in outpatients in two centres (Atlanta (N=979) and Los Angeles (N=913)); Additional 767 Spanish speakers;  Convenience | English and Spanish; analysed separately | TOFHLA  (0-59 inadequate; 75-100 adequate) | Health status;  Access to and use of health services | Results for total English speaking patients: Inadequate health literacy patients more likely to self-report health as poor compared with adequate health literacy (Adjusted OR 2.19, 95% CI: 1.34-3.59).  Atlanta (N=979) Greater likelihood of hospitalisation for inadequate vs. adequate health literacy (Adjusted OR 1.53, 95%CI: 1.39 to 2.76) No differences in ambulatory care use after adjustment. |
| Bennett I (2006), US[21] | Cohort; Focus groups and clinical records | 202 postnatal women hospital inpatients; Sequential convenience | English (assumed) | REALM  (<=6th grade; 7th grade +) | Access to and use of health services | No significant difference between women with low and high health literacy in beginning prenatal care after the first trimester (70% v. 59%; p=.257); or scoring on the Adequacy of Prenatal Care Utilisation Index (58% v. 49%; p=.341). |
| Brez SM (1997), Canada[33] | Multiple case study; semi- structured interviews and participant observation | 8 adults in community college literacy program;  Self referral or reputational referral to study | Ability to speak English fluently | Placement in literacy program  (No comparison) | Access to and use of health services | Participants had found uninformed consent an issue; and reported feelings of powerlessness and vulnerability. They balanced risks of disclosure of literacy difficulties to healthcare staff against risks of non-disclosure. |
| Davis TC (2006), US[23] | Cross-sectional; Face to face interviews | 400 women attending family planning clinic; Convenience | English (assumed) | REALM  (<=6th grade; 7th-8th grade; 9th grade+) | Knowledge | Knowledge of contraception and oral contraception generally poor but higher in groups with higher health literacy level. Poor understanding of side effects of oral contraception or multiple missed pills regardless of health literacy level. |
| DeWalt D (2007), US[38] | Retrospective cohort; Face to face interviews | 150 parents of child hospital outpatients with asthma; Convenience | English main language | REALM  (<9th grade; 9th grade +) | Self-management of health problems;  Knowledge | Children of parents with low health literacy used more frequent (mean days per week 2.7 vs.1.5, p=.01) and greater weekly dosage (mean 6 vs. 3 doses, p<.03) of rescue medication; less controller medication but difference NS; had greater incidence of hospitalisation (adjusted IRR 4.6; 95% CI 1.8 to 12); days missed from school (adjusted IRR 2.8; 95% CI 2.3 to 3.4) and emergency department visits (adjusted IRR 1.4; 95% CI 0.97 to 2.0). Low literacy associated with less parental asthma-related knowledge (14 vs 16 correct out of 20, p<.001). |
| Gazmararian JA (1999), US[43] | Cross-sectional; Face to face interviews in home | 406 community women enrolled in health care community plan; Random sample; Phone call to recruit | 97%  English (assumed) | s-TOFHLA  (<80% correct; 80%+ correct) | Knowledge | Women with lower health literacy more likely to want to know more about birth control (OR 2.2, 95% CI: 1.1-4.4) and more likely to have incorrect knowledge of when they were most likely to become pregnant (OR 4.4, 95% CI: 2.2 – 9.0). |
| Graham J (2007), US[37] | Cross-sectional; Face to face interviews and pharmacy records | 87 HIV- infected clinic outpatients; Sequential convenience | English (assumed) | REALM  (<9th grade; 9th grade +) (Total score) | Self-management of health problems;  Knowledge | Higher health literacy associated with greater likelihood of 95%+ adherence to medication (64% vs. 40% p<.05) over 3 months. Three out of 7 mistaken beliefs associated with health literacy; Adherence norms associated with adherence independent of literacy and did not mediate the relationship between health literacy and adherence. |
| Hicks G (2006), US[42] | Cross-sectional; multiple choice questionnaire interview | 372 patients offered an HIV test by their providers at urgent care centre; Convenience | Ability to read English | REALM  (<=6th grade; 7th grade +) | Knowledge | HIV/AIDS knowledge strongly associated with patients' health literacy (ANOVA p<.001, adjusted R^2^=0.1354). |
| Kalichman S (1999), US[36] | Cross-sectional; Face to face interviews | 318 HIV positive adults living in community; Provider referral; Word of mouth and self-selection through flyers | Fluent English speakers | Adaptation of TOFHLA reading comprehension scale  (<=85% correct; 86%+ correct) | Self-management of health problems | Those with lower health literacy more likely to be non-adherent (at least one dose missed over previous 2 days) to highly active antiretroviral therapy (HAART) (OR 3.9, 95% CI: 1.1-13.4). |
| Kalichman S (2000), US[27] | Cross-sectional; Questionnaire (oral if required) and face to face interviews | 339 HIV positive adults living in community; Provider referral; Word of mouth and self-selection through flyers | Fluent English speakers | TOFHLA reading comprehension scale (Cut-off 80% correct) | Health status; Access to and use of health services;  Knowledge | HIV-AIDS patients with lower health literacy had lower likelihood of undetectable viral load (p<.01); lower CD4 cell counts (p<.05) and more likely to have CD4 cell count <300 cells/mm^3^ (p<.04); perceived their health as poorer (p<.03); and less likely to be on antiretroviral medication (p<.02). Lower literacy associated with poorer knowledge of CD4 count (p<.05) or viral load (p<.01) and poorer AIDS-related disease and treatment knowledge (p<.01). HIV-AIDS patients with lower health literacy less likely to know their CD4 cell count (AOR 1.9, 95% CI 1.1-3.5); less likely to understand meaning of CD4 cell count (AOR 1.7, 0.9-3.3); less likely to know their viral load (AOR 2.3, 95% CI 1.3-3.9); less likely to understand meaning of viral load (AOR 2.2, 95% CI 1.1-4.8) Difference in 3+ hospitalisations for HIV NS after adjustment for time since testing positive. No significant difference in diagnosis with neurological conditions. Those with lower health literacy more likely to believe medical care not good but difference NS. |
| Kalichman S (2000), US[44] | Cross-sectional; Face to face interviews | 294 HIV positive adults living in community; Provider referral; Word of mouth and self-selection through flyers | Fluent English speakers | TOFHLA reading comprehension scale (<=85% correct; 86%+ correct) | Emotional responses | HIV-AIDS patients with lower health literacy had greater symptoms of affective depression (F(1, 290)=16.63, p<.01) but endorsed less negativistic thinking (F(1, 290)=10.89, p<.01). Lower health literacy endorsed feeling more devastated (F(1, 94)=5.84, p<.02), less optimistic (F(1, 94)=8.10, p<.01), afraid (F(1, 94)=4.79, p<.03) and adopting several avoidant coping strategies (p<.05) when presented with scenario of increased viral load. Lower literacy less social support than higher literacy (F(1, 290)=6.95), p<.01). |
| Kalichman S (2000), US[35] | Cross-sectional; Questionnaire (oral if required) | 294 HIV positive adults living in community; Provider referral; Word of mouth and self-selection through flyers | Fluent English speakers | TOFHLA reading comprehension scale  (Cut-off 80% correct) | Access to and use of health services; Knowledge | HIV-AIDS patients with lower health literacy less likely to report undetectable viral load (OR 2.0 95% CI: 1.1-8.1); more likely to visit doctor at least once a month (OR 2.3 95% CI: 1.2-4.4); less likely to say doctor asked their opinion about treatment (χ^2^=10.52, df=3, p<.01) or explained things so they could understand (χ^2^=7.91, df=3, p<.05). Differences in knowledge of CD4 cell counts and viral load NS. Of those who knew health markers, lower literacy less likely to understand meaning of CD4 cell count (AOR 2.5 95% CI:1.2-5.4)or viral load (AOR 3.4 95% CI: 1.3-9.1). Lower literacy more likely to believe anti HIV medication makes patients less likely to transmit HIV to sex partners (AOR 3.0 95% CI: 1.4-6.3) and safe to have unprotected sex if undetectable viral load (AOR 5.8 95% CI: 2.2-15.5); more likely to state easier to relax about unsafe sex (AOR 6.0 95% CI: 2.6-13.6) and report practising more unsafe sex (AOR 3.4 95% CI 1.5-7.5) because of new treatments. |
| Kaufman H (2001), US[32] | Cross-sectional; Face to face interviews | 61 patients at public health clinic; Convenience | English first language | REALM  (<9th grade; 9^th^ grade+) | Health promoting/risk behaviours; | Mothers with lower health literacy less likely than those with higher health literacy to initiate and sustain breastfeeding for first two months (23% vs. 54%) but difference NS. Breastfeeding for two months associated with health literacy but did not reach statistical significance. |
| Lindau ST (2006), US[24] | Cohort; Face to face interviews and clinical records (patients). Self administered questionnaires (physicians) | 68 female attending ob/gyn continuity of care clinic with abnormal pap smear diagnosis; Convenience | 4% Spanish speaking | REALM  (<9th grade; 9th grade +) | Self-management of health problems | High level of agreement between physicians’ subjective assessment and objective measurement of health literacy (Κ=0.43, p=0.0006). Patients with inadequate health literacy less likely to follow up after abnormal pap smear within one year but difference NS (Adjusted OR 3.8 . 95% CI: 0.8-17.4). Subjective physician assessment of patient health literacy significant predictor of follow up within a year (Adjusted OR 14, 95% CI: 3 - 65) but not of time to follow up. |
| Moon RY. (1998), US[25] | Cohort; Face to face interviews and clinical records; Follow-up telephone interviews | 543 parents accompanying children for acute care outpatient visits across five sites; Convenience | English first language | REALM  (Total score) | Access to and use of health services;  Self-management of health problems; | In multiple regression analysis, parental health literacy level (REALM score) did not correlate with use of preventive services Parental understanding of child’s diagnosis, medication name, instructions, purpose or ability to administer medication not associated with health literacy. REALM score correlated significantly with parental perception of how sick their child was (r=-.086 p<.01). |
| Paasche-Orlow MK (2005), US[31] | Cross-sectional; Face to face interviews | 423 women in prison; Convenience | English speaking | REALM  (<=6th grade; 7th-8th grade; 9th grade +) | Health promoting/risk behaviours; | No association between health literacy level and HIV risk behaviour. |
| Rosenthal MS (2007), US[34] | Cross-sectional; Face to face interviews | 157 caregivers of well-child outpatients pediatric residency clinic; Convenience | English speaking | REALM  (<9th grade; 9th grade +) | Access to and use of health services | Caregivers with lower health literacy more likely to report family centred care (79% vs. 61%, p=.03) and helpfulness and confidence building (79% vs. 57%, p=.01). No significant difference by health literacy level in psychosocial issues, safety issues or anticipatory guidance. |
| Ross LA (2001), UK[39] | Cross-sectional; Clinical measurement | 78 families attending diabetic clinic; Convenience | English first language | NART  (Error score converted to IQ) | Self-management of health problems | In children with type 1 diabetes, HBA_1c_ correlated with mothers’ reading scores (r=0.28, p=0.01). |
| Rutherford J, UK | Cross-sectional | Females attending family planning clinics | English first language | REALM | Self care: preventive health | Functional health literacy related to sexual behaviour and knowledge. Low literacy less knowledge and more risky behaviour |
| Rutherford J (2006), UK[22] | Cross-sectional; Face to face interviews | 505 female family planning clinic attendees; Convenience | English first language | REALM scores matched to UK reading age  (12-14 yrs;  15 yrs +) | Health promoting/risk behaviours;  Knowledge | Women with lower health literacy more likely to have been aged under 16 at first sexual intercourse (57.9% vs. 41.6% p<.001); less likely to have used contraception at first sexual intercourse (83.7% vs 89.8% p<.05); and to have had two or more partners in previous 6 months (19% vs. 9.5% p<.002). Difference in planned or unplanned pregnancies, previous use of emergency hormonal contraception and no. of sexual partners in previous four weeks NS. Women with lower health literacy less likely to identify several STIs (χ^2^ = 89.3, df=1 p<.001); to know how STIs are transmitted (giving oral sex χ2= 31 df=2 p<.001; receiving oral sex χ^2^= 81.8 df=2 p<.001; anal sex χ^2^= 92.1, df=2 p<.001) or to know most fertile time of menstrual cycle (χ^2^=38.3, df=1 p<.001). |
| Sharp LK (2002), US[29] | Cross-sectional; Face to face interviews and clinical records | 130 clinic outpatients referred for colposcopy after abnormal pap testing; Sequential convenience | English speaking | REALM  (<9th grade; 9th grade +) | Health promoting/risk behaviours;  Emotional responses | Women with higher health literacy reported higher number of risk factors for cervical cancer (2.3 vs. 1.8, p<.01) but variation in individual risk. Those with higher health literacy more likely to be using oral contraception (34.7% VS. 12.1%, p<.01) and to have had 5 or more sexual partners (51.4% vs. 25.9%, p<.01). Women with lower health literacy higher parity despite similar age (1.9 vs. 1.2, p<.05). Health literacy not associated with intercourse ≤18 years old or sexually transmitted disease other than HPV. Lower health literacy associated with excessive levels of distress among women at high risk for developing cervical cancer (43% vs. 25%, p<.05). |
| Weiss BD (1992), US[28] | Cross-sectional; Questionnaire (Sickness Impact Profile) | 193 adult learners; Stratified random sample by level of reading | English spoken at home as a child | Test of basic adult education  (<=4th grade; 5th grade+) | Health status | Low Iiteracy and poor health status independently associated. Low literacy group had higher physical health score (Adjusted mean 6.2 vs. 2.3, p<.002) and lower psychosocial health score (Adjusted mean 15.4 vs. 8 p<.02) both indicating poorer health on Sickness Impact Profile. |
| Wolf MS (2005), US[41] | Focus groups | 25 HIV- infected clinic outpatients; Convenience from a previous cohort | English (assumed) | REALM  (<9th grade; 9th grade +) | Knowledge | Patients receiving treatment for HIV infection found it difficult to define adherence; required visual cues to identify medication; and had short recall time frame for missed doses (≤ 3 days). Those with low health literacy skills may find it difficult to respond to existing medication adherence questionnaires. |
| Yin HS (2007), US[40] | Cross-sectional; Face to face interviews | 181 paediatric emergency room attenders; Convenience | English and Spanish; analysed separately | TOFHLA  (0-74 inadequate/ marginal; 75-100 adequate) (Total scores) | Self-management of health problems; Knowledge | In adjusted analysis, lower reading comprehension and numeracy scores in parents and caregivers were associated with lack of knowledge about weight based dosing (AOR 2.0; p=.03) and reported use of nonstandardised dosing instruments; (AOR 2.4; p=.007) inadequate/marginal health literacy associated with lack of knowledge of weight based dosing for children’s medication (AOR 2.3; p=.03). |
